# Spatial gene expression quantification: a tool for analysis of *in situ *hybridizations in sea anemone *Nematostella vectensis*

**DOI:** 10.1186/1756-0500-5-555

**Published:** 2012-10-05

**Authors:** Daniel Botman, Jaap A Kaandorp

**Affiliations:** 1 Section Computational Science, University of Amsterdam, Science Park 904, 1098 XH, Amsterdam, The Netherlands

**Keywords:** *Nematostella vectensis*, Gene expression quantification, Gene network modelling, Embryonic development, Embryo morphology

## Abstract

**Background:**

Spatial gene expression quantification is required for modeling gene regulation in developing organisms. The fruit fly *Drosophila melanogaster* is the model system most widely applied for spatial gene expression analysis due to its unique embryonic properties: the shape does not change significantly during its early cleavage cycles and most genes are differentially expressed along a straight axis. This system of development is quite exceptional in the animal kingdom.

In the sea anemone *Nematostella vectensis* the embryo changes its shape during early development; there are cell divisions and cell movement, like in most other metazoans. *Nematostella* is an attractive case study for spatial gene expression since its transparent body wall makes it accessible to various imaging techniques.

**Findings:**

Our new quantification method produces standardized gene expression profiles from raw or annotated *Nematostella in situ* hybridizations by measuring the expression intensity along its cell layer. The procedure is based on digital morphologies derived from high-resolution fluorescence pictures. Additionally, complete descriptions of nonsymmetric expression patterns have been constructed by transforming the gene expression images into a three-dimensional representation.

**Conclusions:**

We created a standard format for gene expression data, which enables quantitative analysis of *in situ* hybridizations from embryos with various shapes in different developmental stages. The obtained expression profiles are suitable as input for optimization of gene regulatory network models, and for correlation analysis of genes from dissimilar *Nematostella* morphologies. This approach is potentially applicable to many other metazoan model organisms and may also be suitable for processing data from three-dimensional imaging techniques.

## Findings

### Introduction

Spatial gene expression assays are a substantial tool for verifying predicted regulatory interactions and for predicting properties of missing components in a regulation network [1,2]. Still, their largest potential is in inferring numerical models of regulatory interaction networks, which is demonstrated for the embryonic development of fruit fly *Drosophila melanogaster*. To perform accurate simulations, the spatial gene expression patterns are digitally quantified and formatted to consistent profiles.

**Figure 1:**
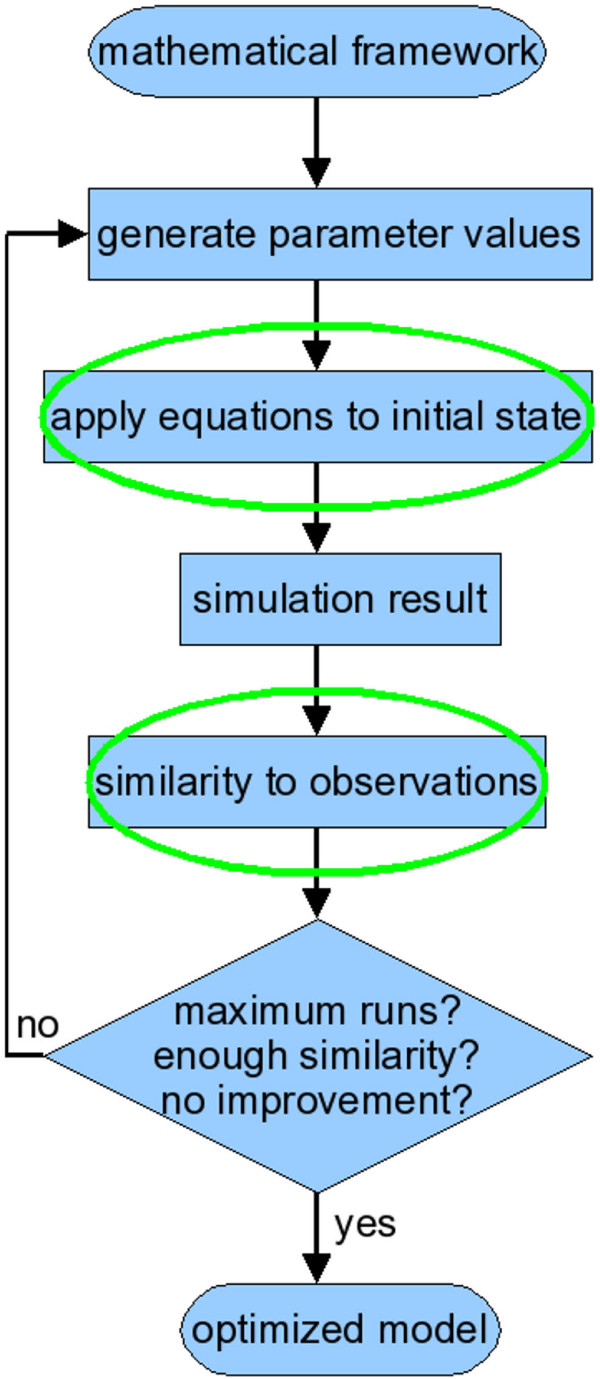
**Overview of the modeling cycle. **The modeling cycle starts with a framework of general mathematical equations. Initial parameter values are randomly generated or manually provided. These values are substituted into the general framework to define a specific set of equations. The equations are applied to the initial state of the system (usually derived from measurements) and produce intermediate and final states. These simulated states are compared to reference data and their similarity is determined. New parameter values are generated and new simulation runs are performed repeatedly, while stopping conditions are tested after each run (such as a maximum number of runs, a target similarity or a lack of improved similarity after multiple runs). As soon as a stopping condition applies, the cycle is terminated and the set of parameter values that results in the closest match with the observations is the optimized model. The steps that require quantitative data are encircled.

In systems biology, computational tools have become indispensable for deriving and validating gene regulation networks [3]. In data-driven modeling, parameter estimation can determine which set of rules represents the best network model to match a set of observations. Figure 1 shows an overview of the modeling cycle. First, a general mathematical framework is selected. The gene circuit model [4,5] (which is derived from the connectionist model [6]) is a convenient formalism that does not require knowledge about interactions or their mechanisms. The actual modeling starts with choosing parameter values for the general equations; the initial parameters are defined by the user or generated by an algorithm. The resulting equations are applied to the concentration profiles at the first timepoint, derived from experimental data. This simulation produces concentration profiles at various timepoints. The simulated profiles are compared with observed expression profiles and the similarity is calculated with a predefined fitness measure. An optimization algorithm then picks new parameter values and starts a new simulation cycle. The optimization process ends when a stopping criterion is reached, such as a fixed time duration or number of cycles, a satisfactory fitness or a series of cycles without significant fitness improvement. The output is the set of parameters that define the equations which represent the observed gene expression profiles best.

In any modeling approach, meaningful results require accurate reference data to initiate the simulation and to evaluate the simulation output. For gene regulation networks, this means that gene expression patterns should be quantified in a consistent manner. A digital quantification procedure has been created and validated for the fruit fly [7].

Gene expression in *Drosophila* is quantified along a straight line during superficial cleavage. In this stage, nuclei are dividing within a single cell membrane and the embryo’s outline does not change significantly. In most other animals however, nuclear division is coupled to cell division during cleavage and the early embryo displays rapid cell movement and morphological changes. This is why we developed a method for gene expression quantification that accounts for a complex and changing embryo morphology.

Over the past decade, *Nematostella* has become an important model organism in the field of evolutionary developmental biology [8]. As a research object, the animal is easy to culture and its small size and transparent body wall make it suitable for all kinds of microscopy. Subsequent gene expression studies and the sequencing of the genome have shown that *Nematostella*, curiously, shares more genes with humans than either *Drosophila melanogaster* or *C. elegans*[9]. Much work on *Nematostella* has been dedicated to the genetic regulation of development [10].

**Figure 2:**
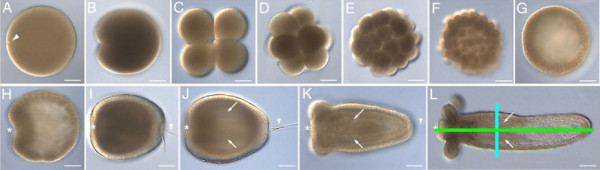
**Various stages of *Nematostella vectensis *embryonic development. **Development stages from egg to polyp are shown, with the oral pole to the left in panels H-L (indicated with an asterisk). **A**) Unfertilized egg, with the female pronucleus visible near the cell membrane at the oral pole (arrowhead). **B**) The first cleavage (1–2 hpf) occurs at the oral pole of the fertilized egg. **C-F**) Cleavage stages, with the 4-cell stage often arising after the first two cleavages finished simultaneously (C). **G**) Cleavages result in a hollow sphere of cells called a coeloblastula (10–20 hpf). **H**) Invaginating cells at the oral pole mark the beginning of gastrulation (24 hpf). **I**) The early larva (72 hpf) displays a doubly layered body wall and swims with the aboral end forward (arrowhead indicates the apical organ). **J**) The late larva starts to elongate and to form two primary septa (arrows). **K**) Early polyp, with four tentacle buds around the mouth at the oral pole. **L**) Juvenile polyp; the primary and secondary axes are indicated with a green and a blue line, respectively. Scale bars are 60 μm in panels A-H and 90 μm in panels I-L. Development times in hpf = hours past fertilization (original image from [11], with development times estimated from [13,29]).

The early developmental stages of *Nematostella vectensis* are displayed in Figure 2 (adapted from [11]). The *Nematostella* body wall consists of an outer cell layer (ectoderm) and an inner layer (endoderm). We are primarily concerned with the invagination process called gastrulation, when the presumptive endoderm moves inwards and covers the ectoderm. The side of invagination is the location of the future mouth and is therefore referred to as the oral pole; the opposite side is the aboral pole. *Nematostella* displays bilateral symmetry: the line between the oral and aboral ends is the primary axis, while the line through the primary septa defines the secondary axis (the green and blue lines, respectively, drawn on Figure 2L).

In this paper we discuss a geometric method for extracting quantitative spatio-temporal gene expression data from *in situ* hybridizations in the sea anemone *Nematostella vectensis*. We measure gene expression during gastrulation using a gene expression quantification tool developed by de Jong [12]. We show in some preliminary results how this information can be analysed in a cluster analysis.

### Results

#### Gene expression quantification

**Figure 3:**
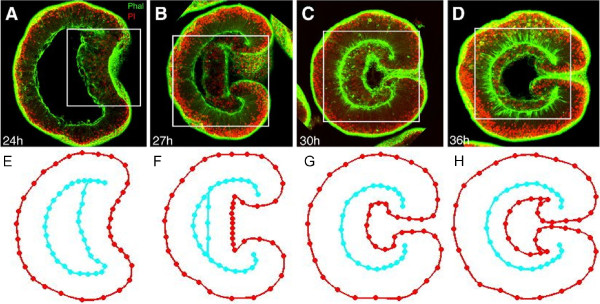
**Designing graphical embryo geometries from confocal microscopy images of *Nematostella vectensis. *AD**) Fluorescent *Nematostella *embryos in subsequent stadia of gastrulation stained with phalloidin (green) to label actin and propidium iodide (red) to label nuclei (taken from [13]). Hours of development times are indicated in the lower left corner of each panel. **EH**) Average geometries constructed from shown and additional confocal images [12].

Microscopy images accentuate cell layer outlines of *Nematostella* embryos stained for nuclei and filamentous actin (Figure 3A-D, copied from [13]). The latter is bound to the membrane as part of the cell cortex, so it highlights cellular shapes. From these images, average embryo morphologies have been derived for various stages of gastrulation (Figure 3E-H). An embryo geometry is derived from a confocal microscopy image (such as Figure 3A) by placing nodes on the cell layer boundaries. Node locations from multiple (2 to 5) geometries are averaged to obtain an average embryo geometry (such as Figure 3E). This averaging reduces the effect of local irregularities, as the average geometry is supposed representative for embryos of a particular age. Strategic points are picked to define a shape suitable for interpolation with the geometry in the subsequent stage. Interpolation of these average morphologies results in a continuous range of embryo morphologies.

In the following example, these graphical shapes are applied to quantify gene expression patterns with the GENEXP program (Additional file 1). Published *Nematostella* gene expression images are collected in the CnidBase [14] and Kahi Kai [15] databases, and Kahi Kai also contains expression images outside journal publications. Still, many *Nematostella* expression pictures are found in publications outside these databases.

##### Example 1: a 1D expression profile from a symmetrical pattern

**Figure 4:**
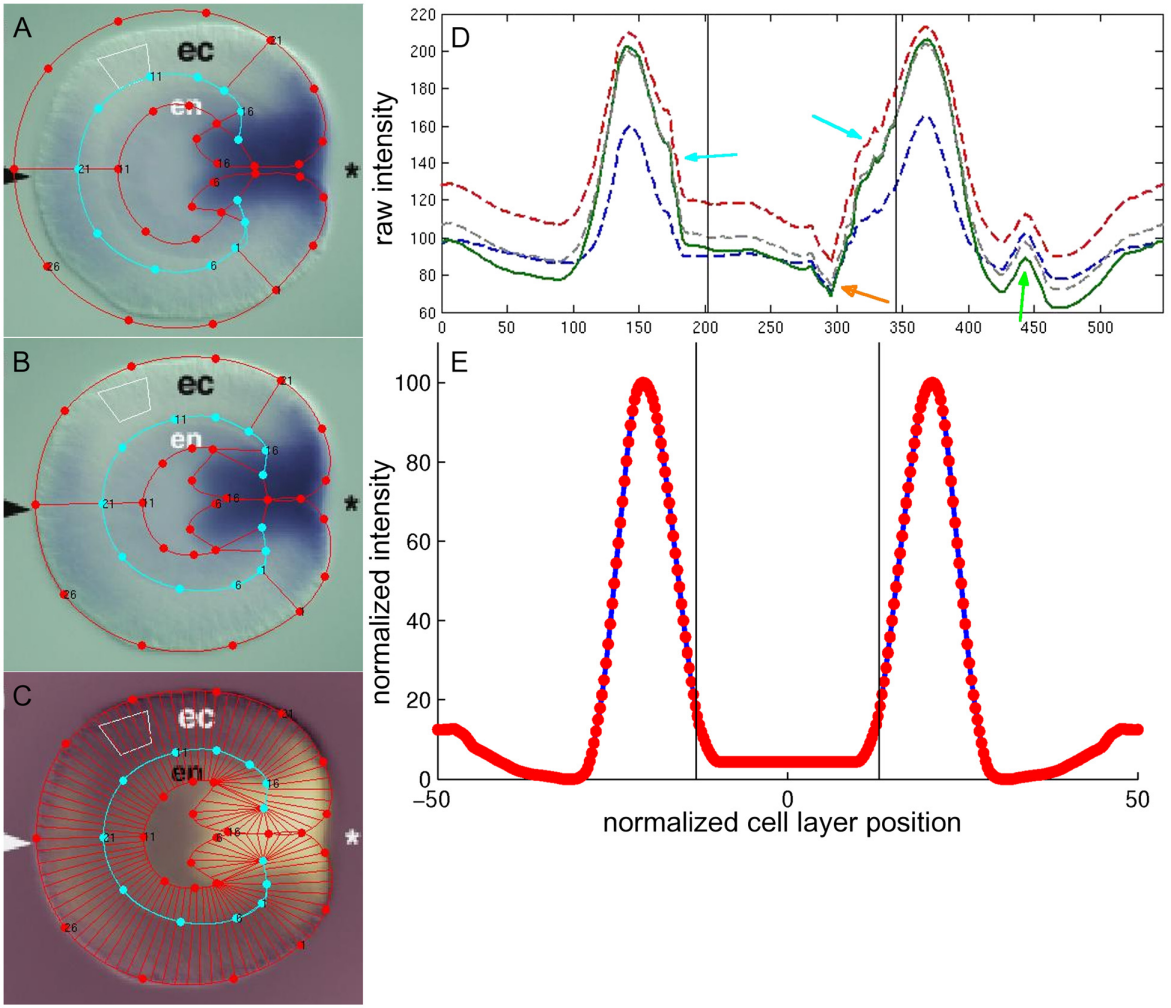
**Steps in obtaining a quantified gene expression profile from a *Nematostella in situ *hybridization. A**) An unaltered embryo geometry (chosen from a list of interpolated geometries) is projected on an *in situ *hybridization of *Nvnos2 *at the end of gastrulation (original image from [16]). In the original image, the arrowhead highlights gene expression in the aboral ectoderm and the asterisk denotes the future mouth. Endoderm and ectoderm are labeled ‘en’ and ‘ec’, respectively. **B**) The points in the graphical geometry are manually dragged over the cell layer boundaries of the observed *Nematostella* embryo. **C**) After applying a color inversion, the embryo is decomposed into parallel sections along the cell layer. **D**) For each section in figure 4C, the average red, green, blue and greyscale intensities are plotted in an expression profile as a function of its position along the cell layer. The segment on the aboral end of the decomposition corresponds to 0 on the horizontal axis; from here, the decomposition proceeds counterclockwise along the cell layer. After crossing the endoderm-ectoderm boundaries (which correspond to the vertical black lines in the plot), the decomposition again reaches the aboral end, corresponding to the right side of the horizontal axis in the intensity plot. Arrows highlight artefacts (see example 1 in the main text). The main profile is plotted as a solid graph. **E**) After vertically and horizontally shifting the main profile from panel D, artefacts are removed, noisy regions are smoothed and the profile is symmetrized. On the horizontal axis, the endoderm center lies at normalized cell layer position 0 and the ectoderm center is normalized at −50 and +50, while the maximum intensity is normalized to 100 on the vertical axis.

Figure 4A (adapted from [16]) displays an *Nvnos2 in situ* hybridization in a late gastrula. The transcripts were hybridized with digoxigenin-labeled RNA probes. The published image is overlaid with the most similar morphology. The graphical geometry is then adjusted to the outline of this particular embryo by dragging the nodes to their final location (Figure 4B). The nodes are connected by a curve (about 10^5^ points) calculated with cubic spline interpolation [17]. The geometry is automatically decomposed into parallel segments along the cell layer and color intensities are measured for all segments. The decomposition is performed by dividing the outer curve into sections with a user-defined length and calculating the nearest point on the inner spline for each boundary. This is repeated for the inner curve at sections with large gaps. The decomposition is finished by smoothening the boundary points on both curves to obtain segments that are more uniformly spaced. For the expression profile the red, green and blue color intensities of all pixels enclosed within a segment are averaged. These average intensities are plotted against the position on the line through the segments’ centers. To make an increasing intensity indicate an increase in concentration, the colors are inverted in Figure 4C and the corresponding intensities along the cell layer are displayed in Figure 4D.

The graph in Figure 4D contains features that do not represent the actual transcript concentration. The “en” and “ec” annotations cause a trough (orange arrow) and a ridge (green arrow), respectively, while an imperfect decomposition has shifted the peaks with regard to the center and introduced some noise (blue arrows). Moreover, the nonuniform background and nonsymmetric lighting cause an asymmetric baseline. The profile is exported to an editor for additional processing to correct for these features. To remove artefacts caused by annotations, the user selects this section of the graph and can choose among linear interpolation, cubic spline interpolation, piecewise cubic Hermite interpolation [17] and replacement with a specified constant. Noise from erroneous decomposition is smoothened with a lowpass filter (with filter coefficients equal to the reciprocal of the span), known as ‘moving average’ [18]. The graph is lowered with a constant value to subtract the average background and regions without observed expression are put to zero. Both halves are averaged to cancel nonsymmetric influences. The final expression profile is plotted in Figure 4E.

#### Three-dimensional expression pattern reconstruction

For expression patterns that are radially symmetrical around the primary axis, a one-dimensional profile is a complete description. However, most signaling pathways involve genes that are asymmetrically expressed along the secondary axis. A three-dimensional representation is required to fully define the expression pattern of these genes. Depending on the data available for a gene expression pattern, a suitable method is determined for the approximation of this 3D pattern.

**Figure 5:**
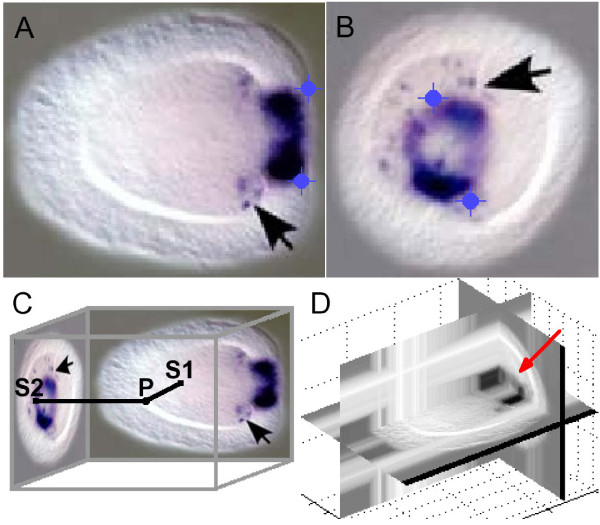
**A three-dimensional array constructed from perpendicular *Nematostella *embryo views.AB**) *NvFoxB* expression in the early larva appears focused on two spots around the oral pole (original images from [19]). Reference points are indicated as blue crosshairs on lateral (**A**) and oral (**B**) views to shift and scale the oral image’s height. In the original images, the black arrows highlight isolated expression in some endodermal cells. **C**) A coordinate sweep is performed over the three-dimensional array and the corresponding points *S1* and *S2* on the lateral and oral images, respectively, are determined. The point with minimal signal is selected for the new array **P**. Because the signal is darker than the background, the minimal signal corresponds to the maximal intensity, as expressed in the algorithm in figure 6A. **D**) The greyscale intensity is displayed in three perpendicular planes of the 3D gene expression array. The main features of the expression pattern in panels A and B can be identified in this representation (red arrow).

**Figure 6:**
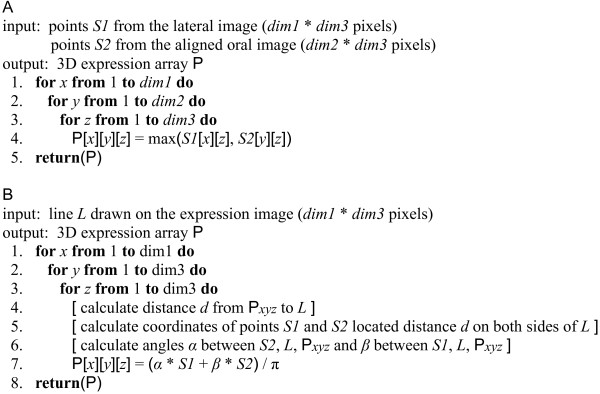
**The pseudocodes for 3D expression construction. A**) The pseudocode for constructing a 3D expression array based on two aligned perpendicular embryo views. **B**) The pseudocode for calculating a 3D array from a single expression image with a manually drawn line dividing the expression domain.

**Figure 7:**
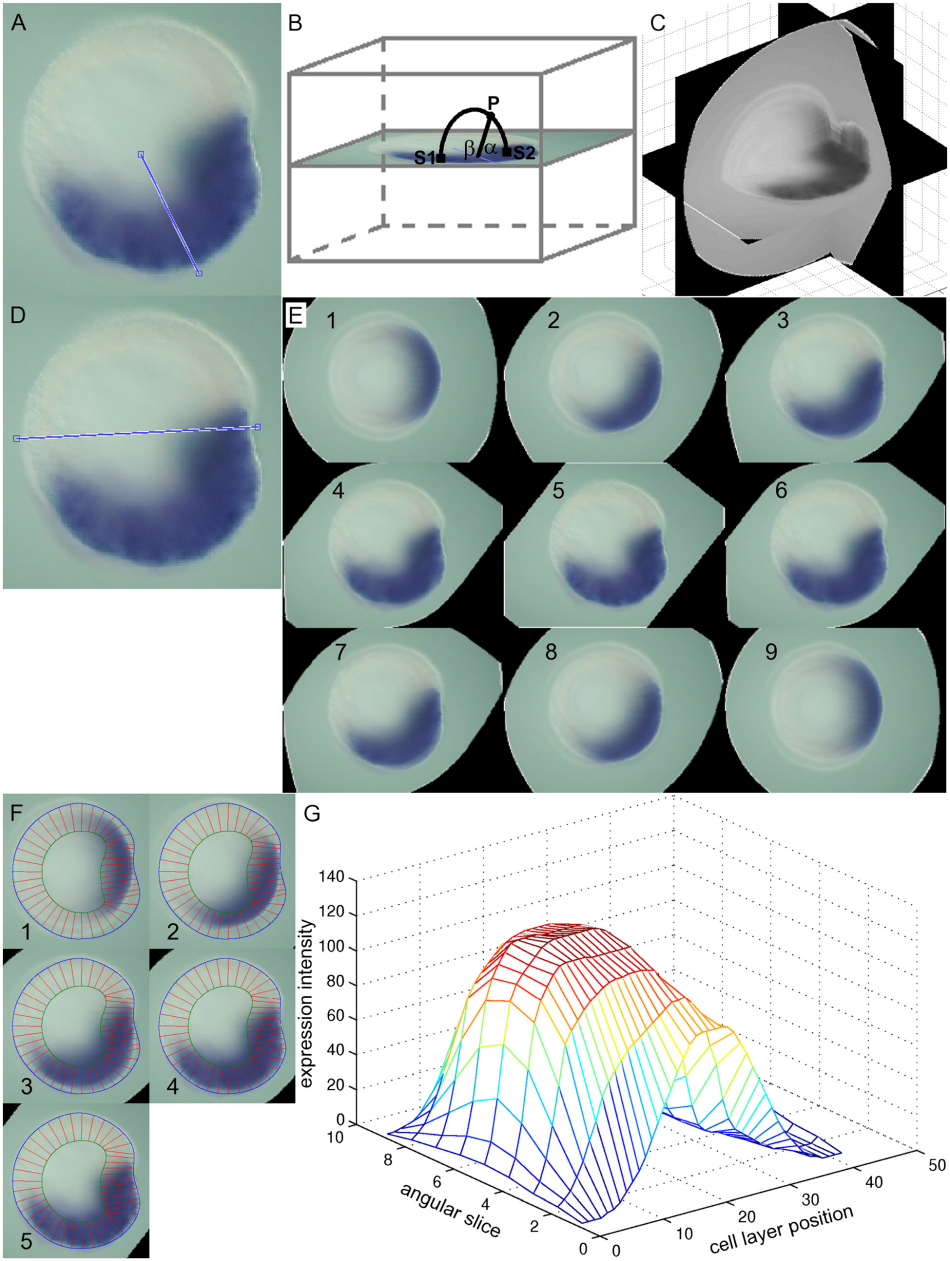
**A 2D quantified expression profile from a single *Nematostella *gene expression image. A**) At the start of gastrulation, the gene *Nvvas2 *is expressed roughly symmetrically with respect to the blue axis drawn (image from [16]). **B**) For all points of the 3D array **P**, the base points *S1 *and *S2* of the arc centered on this axis are determined. The intensity is calculated as the weighted average of these base points, as expressed in the algorithm in figure 6B. **C**) The greyscale intensity is displayed in three perpendicular planes of the 3D gene expression array. The horizontal plane is the truncated original. **D**) The primary axis is indicated on the original image. **E**) From the 3D expression, slices are calculated at equally incremented angles through the primary body axis. **F**) The original embryo geometry is overlaid on the first five pattern slices from figure 7E (the last four are identical in reverse order). As expected, the embryo geometry does not match the expression outlines. The geometry is not adjusted to the expression, because the expression representation is not meant to approach the embryo’s shape. Besides, the expression outline outside the central *x,y* plane is too blurred for a precise geometry extraction. (The distortion is more obvious with a dark background, as seen in Figure 5D.) **G**) After processing each 1D expression profile derived from the decomposed angular slices, the complete two-dimensional expression array is plotted as an intensity landscape. The cell layer position follows the segments in the decomposition in counterclockwise direction, starting and finishing at the aboral end. The angular slice designations correspond to the number labels in panel E. Processing of one-dimensional profiles is done in a similar fashion as in example 1 (main text).

If a set of perpendicular expression images shows that an expression pattern is not radially symmetrical, then a 3D representation is constructed by mixing both images into a three-dimensional array (Figure 5C) with the TWOVIEWS program (Additional file 1). With points *S1* on a lateral view in the *x,z* plane and points *S2* on an oral view in the *y,z* plane, the points **P** in the 3D array are defined by the algorithm in Figure 6A. Otherwise a radial expression pattern symmetry is assumed, and a 3D representation is created by smoothly averaging pairs of points on the image along circular arcs about an approximate axis of symmetry (Figure 7B) with the TWOVIEWS program (Additional file 1). The pseudocode for this operation is displayed in Figure 6B.

##### Example 2: a 3D representation from perpendicular embryo views

Figure 5A,B (adapted from [19]) shows the expression pattern of gene *NvFoxB*, which is concentrated on two spots on opposite sides of the oral end. Two sets of reference points are picked to scale and align both pictures. The height of the oral image is adjusted to match these points in the lateral image.

Elements for the 3D array that represents the volumetric expression pattern are calculated as the minimum expression from the associated pair of pixels (Figure 5C). A greyscale visualization of this array is displayed in Figure 5D. Two domains appear in the oral region as expected.

The 3D array is an intermediate step in the quantification process that is completed for the next example.

##### Example 3: a 2D expression landscape from a single embryo view

Figure 7A (based on [16]) shows the expression of *Nvvas2* in the early gastrula stage. The expression domain covers the embryo’s lower half and its future mouth. A line is drawn on the image that divides the expression domain. Each element in the 3D array is the weighted average of the image pixels at both ends of a circular arc around this line (Figure 7B,C).

This discrete volumetric expression array is not yet suited to be compared to other patterns, because their shapes do not match or their cell layers are located at different Cartesian positions. To arrive at consistent profiles, slices are cut through the primary axis and decomposed. The primary axis is drawn on the original image and slices of the 3D array through this axis are constructed (Figure 7D,E). These slices are overlaid with a geometry that fits the native image (Figure 7F) and through the decomposition procedure described in the one-dimensional example, a set of profiles is produced. These profiles are stacked in a 2D array and displayed as a landscape in Figure 7G.

#### Visualization and clustering

**Figure 8:**
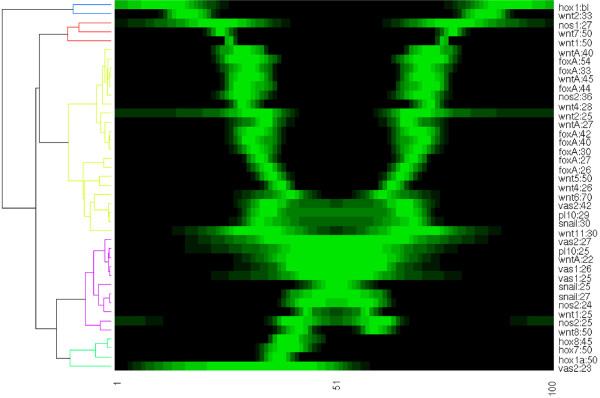
**Synoptic display and clustering of 1D expression profiles of various genes at different development times. **Normalized gene expression profiles are conveniently ordered based on their similarity in spatial intensity distribution. This compact overview contains expression patterns of *hox*, *wnt*, *nanos*, *foxA*, *vasa*, *PL10* and *snail *genes from various sources. Gene name and development time for each profile are displayed on the right in the format {gene}:{hours past fertilization}, except for *hox1* which is in the hollow sphere (blastula, bl) stage and therefore its age cannot be determined from its morphology. The gene expression intensity is scaled from black (no expression) to bright green (maximal expression). The dendrogram on the left shows the correlation between the spatial expression patterns. In this hierarchical clustering, the Pearson correlation coefficient was applied for the similarity measure and average linkage for tree formation. The dendrogram was cut at similarity 0.6 to obtain five groups (blue, red, yellow, purple and green from top to bottom). A detailed description of the function used to display the data and documentation of the algorithms for the correlation is provided at [30].

The profiles are easily interpolated and stored in arrays of equal length. Such a collection of standardized arrays can serve as input for conventional comparison and analysis software. For example, Figure 8 shows 41 1D expression profiles from 20 genes, ordered with hierarchical clustering. In this fashion, a database is conveniently displayed and correlating patterns can be identified at a glimpse.

From the cluster tree, the patterns are divided in three main groups, and five individuals with little similarity. In green, all four asymmetric expression patterns are included. The purple patterns are restricted to the endoderm. The yellow group is the largest, containing genes that are expressed in the presumptive pharynx and mouth. The remaining genes are expressed in ectoderm away from the mouth.

The clustering displayed in Figure 8 might be somewhat artificial as the expression domains clearly overlap. Moreover, some regions are elongating faster during gastrulation than others and even after pinning the estimated endoderm-ectoderm boundary, stationary patterns such as *NvFoxA* seem to migrate. Still, the comparison is very helpful in observing correlations and proposing hypotheses. For example, the three main groups may indicate regulatory modules. More specific, the patterns with broad boundaries belong to embryos in a relatively early stage, indicating a regulatory cascade in which fuzzy domain boundaries are sharpened, comparable to the *Drosophila* gap genes. An extended and systematic set of profiles would enable an inference of the developmental gene regulation network in *Nematostella*, based on the modeling techniques and analyses that established many properties of the *Drosophila* gap gene network [20-22].

### Discussion

Our quantification procedure provides a standardized format for the most diverse spatial visualization techniques. In this paper, hybridized mRNA has been quantified, but the method can be applied to any specific molecular entity. Potential examples include native proteins visualized with antibody staining and overexpressed proteins fused to a fluorescent agent [11,23].

Current limitations arise from the strong assumptions imposed on the images that are used to construct 3D representations. For a rotated pattern, it is formally assumed that only expression in the plane of dissection is visible, while in fact observed expression is not restricted to this plane.

More serious is the requirement that the embryos used for the reverse projection are completely transparent, while the endoderm and aboral ectoderm are hidden on most oral images. Sometimes, only the cumulative signal on the periphery of an expression region is detected, as in Figure 5A (the speckled region at the arrow and on the opposite side).

The embryo is also assumed to be viewed from exactly perpendicular angles, but the sample is often rotated imperfectly. Additionally, slight deformations can occur during rotation, causing small domains with granular expression to overlap improperly and thus to be misrepresented. These issues are observed in Figure 5 as well.

With the advance of direct three-dimensional imaging the volumetric array construction may become superfluous, and these limitations will be removed. Confocal laser scanning microscopy has already been applied to zebrafish [24], *Drosophila*[25] and sea urchin [26] embryos. This method may provide quantitative, spatial expression data for *Nematostella* as well. Conversely, general methods for mapping these data to the embryo’s morphology should be useful for comparison and analysis in these other organisms.

#### Future developments

An integrated method has been presented that combines geometry extraction and gene expression quantification. The basic concept is that gene expression is conveniently measured along the cell layer in a morphology that can be viewed as a continuous sheet of cells. This straightforward approach can be applied generally to embryos across the animal kingdom. As confocal laser scans with high three-dimensional spatial resolution are widely applied, application of this method is not limited to symmetrical body shapes.

We have shown how to extract quantified gene expression profiles from *Nematostella in situ* hybridizations, and how a preliminary comparison and cluster analysis lead to new insights. The next step is to estimate parameters that describe interactions among genes in the *Nematostella* regulatory network. The powerful methods designed for parameter inference [4,5,20] and network analysis [21,22] in *Drosophila* can now be applied to the standardized gene expression profiles of *Nematostella vectensis* and other model species in genetics.

Currently, a database of published images is processed into 1D arrays, annotated with roughly estimated development times based on comparison with high resolution micrographs. This comparison is very subjective, as our designation often differs from the developmental stage originally claimed. Moreover, the embryos change very subtly in the hollow sphere stage (Figure 2G) and between invagination and septa formation (Figure 2I), so timestamps are highly ambiguous. If a gene or a combination of genes is found with continuously changing expression patterns, we can derive labelling protocols to determine the exact developmental time. (Registration techniques like this have already been described and proved useful for *Drosophila*[27].)

Spatial gene expression quantification can be combined with modern quantitative polymerase chain reaction (qPCR) techniques [28]. The absolute total amount of transcripts measured with qPCR coupled to the spatial distribution from quantified *in situ* images should enable the calculation of absolute local mRNA concentrations.

## Availability and requirements

### Project name

BioPreDyn (new bioinformatics methods and tools for data-driven predictive dynamic modelling in biotechnological applications)

Project home page

http://www.biopredyn.eu.

Operating system(s)

Linux, Windows, MacOS.

Programming language

Matlab.

Other requirements

None.

License

None.

Any restrictions to use by non-academics

None.

## Competing interests

The authors declare that they have no competing interests.

## Authors’ contributions

All authors participated in the design of the study, and wrote the manuscript together. DB conducted the analysis of data and simulations, and made the figures. All authors read and approved the final manuscript.

## Supplementary Material

Additional file 1 **Matlab programs: “genexp.m” for cell layer decomposition of gene expression images and profile editing; “oneview.m” for a 2D gene expression array from a single decomposed image; “twoviews.m” for a 2D array from a decomposed image and perpendicular view. **Supporting files: Matlab user interface “genexp.fig” (necessary to run “genexp.m”); directory “embryodata” containing geometry definitions; directory “functions” containing Matlab functions used in the main programs. Matlab script: “stanpro.m” for creating a standardized 1D array from a GENEXP profile figure. Manuals: “readme_genexp.m” for GENEXP program; “readme_oneview.m” for ONEVIEW program; “readme_twoviews.m” for TWOVIEWS program. Click here for file
